# Age-sensitive telehealth group therapy for depression in older adults with and without comorbid anxiety (VISION-AGE): study protocol for a randomized controlled trial in an embedded mixed-methods design

**DOI:** 10.1186/s12888-026-07851-2

**Published:** 2026-02-18

**Authors:** Eva-Marie Kessler, Kristian Kleinke, Jill Viviann Engel, Simon Forstmeier

**Affiliations:** 1 Department of Psychology, MSB Medical School Berlin, Rüdesheimer Str. 50, 14197 Berlin, Germany; 2 Department of Psychology, University of Siegen, Adolf-Reichwein-Str. 2a, 57068 Siegen, Germany; 3 Department of Psychology, University of Siegen, Obergraben 23, 57072 Siegen, Germany; 4 Department of Psychology, MSB Medical School Berlin, Rüdesheimer Str. 50, 14197 Berlin, Germany

**Keywords:** Online psychotherapy, Comorbid depression and anxiety, Digital mental health intervention, Life review therapy, Life planning

## Abstract

**Background:**

There is a necessity to enhance psychotherapeutic treatment approaches for older adults with depression, particularly those exhibiting comorbid anxiety, in both clinical practice and healthcare provision. The aim of the clinical study VISION-AGE is to investigate the efficacy and mechanisms of action of an age-sensitive online group psychotherapy for adults aged 68 + with clinically significant depression (with or without anxiety).

**Methods:**

In a two-arm randomised controlled trial, the hypothesis that enriched life-review therapy, offered in a video-conference group setting, is superior to an active control group, will be tested. Following randomisation into the intervention or control group, all participants attend weekly manual-guided online group sessions for 20 weeks (plus four additional individual meetings) in fixed groups of four participants. In the intervention group, participants are administered psychotherapy, with a focus on promoting self- and developmental regulation strategies (i.e., positive self-perceptions of ageing next to reminiscence, life management and life planning). The active control group takes part in a standardised non-clinical discussion programme promoting peer support and socializing, similar to typical social care services for older adults. The planned sample size is *N* = 250 (*n* = 125 participants per arm). The hypothesized reduction of self-reported depressive symptoms (primary outcome), anxiety as well as clinician-rated depressive symptoms and the hypothesized improvement of psychological well-being (secondary outcomes) will be assessed at baseline, post-treatment and at the 6-month follow-up. Furthermore, qualitative interviews will be conducted with participants of the intervention group in order to gain a more profound understanding of the subjective change processes experienced during the intervention.

**Discussion:**

VISION-AGE explores the potential of online group psychotherapy to address the needs of older adults with clinical depression. Whilst online therapy facilitates convenient access for patients, group therapy format enables therapists to extend their reach. The study will provide practical implications to improve access to and quality of psychotherapeutic care for this under-resourced population. The long-term objective is the development of a transdiagnostic psychotherapeutic programme that is both efficient and cost-effective, and which is accessible and appealing to older patients in regular mental health care.

**Trial registration:**

at The German Register of Clinical Studies (DRKS); 13 November 2025 (Identifier: DRKS00038366; https://www.drks.de/DRKS00038366). Scientific title: Efficacy and modes of action of an age-sensitive telehealth group therapy to reduce clinical depression (with and without anxiety) in older adults (VISION-AGE).

**Supplementary Information:**

The online version contains supplementary material available at 10.1186/s12888-026-07851-2.

## Background

Due to demographic shifts, older adults now make up a growing proportion of the patient population, particularly in light of their substantial medical needs. Approximately one in three older adults has experienced a mental health disorder within the past year, with depression and anxiety being particularly prevalent [1]. A recent review and meta-analysis found a pooled prevalence of 31.7% (95% Confidence Interval [27.90, 35.59]) for depression alone (including mild depression) in the older population worldwide [2] — commonly termed geriatric or late-life depression. Depression in older adults has been shown to be associated with an increased risk of physical morbidity, suicidal ideation, physical and cognitive decline, social isolation, inadequate self-care, and functional impairment [3], resulting in significant welfare costs [4]. Similarly, anxiety disorders are common in late adulthood (affecting around 17% of the population) and are also associated with various cognitive disadvantages, as well as physical and social limitations [5, 6]. Furthermore, there is extensive evidence of frequent comorbidity of depression and anxiety among older people, up to 50% [7], whereby the symptoms often reinforce each other, maintaining negative effects [8].

Substantial deficits remain in the development and implementation of adequate, effective and efficient psychotherapeutic approaches that specifically target older adults, a group that is both under-researched and under-supplied [9–11]. Mental disorders in older adults remain frequently underdiagnosed and inadequately treated across all care settings, often without adherence to established guidelines. Pharmacological treatments predominate, with inadequate medication, side effects, and polypharmacy commonly occurring [12]. The factors contributing to the undersupply are numerous, but include a paucity of appealing treatment offers that target the needs and preferences of patients in later life, a lack of treatment settings that allow mobility-impaired patients to receive treatment in their own home (e.g. home treatment, digital therapy), insufficient referral to psychotherapy by general practitioners and a lack of clinical training programmes for psychotherapists [13].

Our age-sensitive VISION-AGE programme, an enriched life review therapy (LRT) for the primary treatment of depression (with and without anxiety) offered in a videoconference group setting, aims to address and reduce the following research gaps:

1. According to systematic reviews and meta-analyses, standardized interventions for (face-to-face) treatment of depression in older people based on a standardized treatment manual are superior to passive control groups and conventional treatment in terms of their efficacy [14, 15]. Cognitive behavioral therapy (CBT) [16], life review therapy (LRT) [17], and problem-solving therapy [18] have proven most (and similarly) effective. However, when compared to active control groups, pre-post effect sizes are usually significantly smaller (around d = 1.0) [15, 19]. In a very recent randomised controlled trial (RCT) in Germany, a late-life specific CBT intervention (‘CBTlate’) was not superior to non-specific supportive psychotherapy (empathic listening; *N* = 251) [20]. Conversely, this implies that further evidence is needed to validate the specific efficacy of psychotherapeutic treatments for this population and to assess their advantages over cheaper interventions like community-based support groups for older adults. A control group should build on the current state of research in this area and currently, there is no scientifically based standard treatment for late-life depression – especially not in a group setting. When it comes to non-pharmacological services, social group offers correspond most closely to what older adults typically receive under naturalistic conditions in elderly care. Therefore, research needs to test the benefits of group psychotherapy over cheaper interaction offers, like social groups, for this population.
2. Until now, treatment programmes have focused almost exclusively on depression in older patients, since comorbidity is commonly associated with poorer treatment outcomes. Given the high comorbidity of anxiety and depressive disorders [7], a transdiagnostic approach improves the generalisability of the findings and their applicability in outpatient settings.
  Only four preliminary RCTs have assessed the efficacy of psychological interventions for comorbid depression and anxiety disorder [21]. Further research is therefore needed to determine whether a transdiagnostic programme can effectively address symptoms of both depression and anxiety simultaneously.
3. A recent study showed that older patients with depression were more likely than younger individuals to set personal treatment goals aimed at improving their wellbeing and coping with their personal development [22]. Further research is needed to investigate whether psychotherapy that is tailored to age-specific treatment goals, such as improving well-being and encouraging reminiscence, is more effective than regular psychotherapy.
4. So far, life review therapy is the only therapeutic approach to have used findings from lifespan psychology [23], but it has not yet taken all current empirical research findings into account. Reminiscence in older adults, as promoted in life review therapy, has been shown to be prospectively associated with higher levels of life satisfaction and lower levels of depression and anxiety [24]. Other adaptive strategies of self- and developmental regulation (well-researched in lifespan psychology) have not yet been adequately integrated in treatment programmes:
  - There is ample evidence of a significant association between internalised age-related stereotypes and health outcomes [25]. Therefore, it is important to foster a positive self-perception of ageing to avert adverse health-developments and promote well-being.
  - SOC-strategies for life-management (i.e., *selecting* positive and subjectively meaningful aspects of life, *optimising* existing abilities, and *compensating* for deficits) have been shown to be associated with feelings of satisfaction and positive affect in old age [26].
  - Moreover, research shows that future planning tends to decline with age in favour of a stronger focus on the present – although planning is associated with higher levels of well-being and lower levels of depression and anxiety [27].
    Consequently, research should examine whether the augmentation of live-review therapy with these three adaptive strategies (i.e. the enhancement of positive self-perceptions of ageing, the strengthening of SOC-strategies and life-planning) yields therapeutic benefits for older patients.
5. The most recent systematic review on group psychotherapy with older adults [28] included nine controlled studies on the effectiveness of group psychotherapy for late-life depression (defined as 60+). Four of these studies employed a randomised controlled trial design. The systematic review excluded studies involving mental health comorbidities (with the exception of anxiety disorders) and comorbid cognitive impairments. The predominant therapeutic approaches employed were CBT and LRT (also referred to as reminiscence therapy). The extant research demonstrated a significant reduction in depressive symptoms in group therapy, with only one exception. The findings were consistent across diverse settings, encompassing outpatient, inpatient, and nursing facility demographics, as well as individuals across the age spectrum from “young old” to “old old”. Nevertheless, superiority over other psychosocial approaches or active control groups, such as relaxation groups or discussion groups, was not demonstrated in all studies. In addition to the observed reduction in depressive symptoms, a number of the included studies identified favourable outcomes with regard to anxiety, integrity, and life satisfaction. The stability of the achieved improvements remains unconfirmed, as only a limited number of studies have reported follow-up (FU) surveys with equivocal results and in some cases high rates of attrition. Despite the limited evidence, it remains important to investigate the therapeutic benefits of group therapy, as, in contrast to individual therapy, it has a particular potential for this age group. Life-span developmental research has demonstrated that older adults tend to distance themselves in their self-definition from their own age-group [29]. Consequently, the formation of groups has advantageous potential to enhance interpersonal relationships, foster trust, and promote both age identity, which is based on shared experiences and life stages [30], and generational identity, based on being born at the same time [31]. Furthermore, the utilisation of model learning and the collective ego of the group facilitates a more nuanced evaluation of situations, thereby enabling participants to develop more efficacious strategies for addressing typical age-related challenges. In light of this, further research is required to investigate the long-term effectiveness of group psychotherapy involving older adults of the same life stage and cohort.
6. It is evident that there is a significant absence of research in the domain of digital e-mental health interventions for the elderly. A 2023 systematic review [32] identified a total of five RCTs that examined the efficacy of virtual (e.g. telephone, videoconference, or internet-based) non-pharmacological interventions in reducing symptoms of depression in older adults (defined as ≥ 60) – the authors summarise paucity in this research field. In the specific case of videoconference psychotherapy, the efficacy and non-inferiority in comparison to face-to-face therapy for depression and anxiety disorders has been empirically substantiated for younger patients through meta-analyses. For example, in a meta-analysis of video-based CBT treating comorbid depression and anxiety, large effects were found for depressive symptoms (g = 0.79) and anxiety (g = 0.82) [33]. A systematic review of the literature shows that group therapy, in small formats, can be effectively delivered online [34]. The necessity for well-controlled efficacy studies on video-based psychotherapy for older patients is evident, as this type of support could give a larger group of older adults access to psychotherapy, including people with reduced mobility, those living in rural areas and older carers unable to leave home for face-to-face therapy and accompanying travel requirements.

In light of the aforementioned research gaps, the implementation of a video-based group psychotherapeutic programme targeting older adults with depression and comorbid anxiety constitutes a pioneering intervention – particularly in light of the high clinical care demand. In terms of the VISION-AGE programmes’ content, the rationale focuses on improving adaptive forms of self- and developmental regulation strategies. These are outlined as follows: (1) positive self-perceptions of ageing, (2) life review (strengthening reminiscence), (3) life management (training SOC-strategies) and (4) life planning. This approach is embedded within a therapeutic concept that traverses the three biographic ‘time corridors’ past - present - future. The primary therapeutic methods originate from LRT, but were enriched by CBT and therapeutic techniques of Positive Psychology (PP).

In order to develop an efficient psychotherapeutic programme, it is crucial to examine how patients interpret, memorise and realise the psychological input received over the course of a treatment. In this regard, qualitative methodologies provide insight into the individual patient’s perspective on the therapeutic change [35]. Therefore, the present study employs a mixed-methods approach, triangulating quantitative and qualitative methodologies.

In the context of this innovative therapeutic concept, and considering that the manual for the control group represents an adapted extension of an already proven helpful concept [36], the potential benefits and study objectives are the reduction of symptoms of depression, anxiety as well as the increase of psychological well-being and concepts of self- and developmental regulation. No health risks are expected to be associated with data collection or the programme, as all participants will receive support. As participants in both conditions will be dealing with personal issues and each other, temporary mood deterioration may occur. However, given the qualifications, training and supervision of our group therapists (leading the intervention group) and group facilitators (leading the control group), no significant long-term harms or deterioration are expected, even after the programme ends.

### Objectives

The main objective of the present study is to investigate the effectiveness of the age-sensitive telehealth group psychotherapy (VISION-AGE) for older adults with depression (with or without comorbid anxiety) compared to a non-clinical active control condition (a group discussion programme) with regard to depression, anxiety, and psychological wellbeing. Furthermore, the mechanism of action of VISION-AGE will be investigated. Adaptive self- and developmental regulation strategies (defined as positive self-perceptions of ageing, reminiscence, life management, and future planning) are examined as mediators, based on the assumption that strengthening these capacities play a central role in reducing symptoms of depression and anxiety, while enhancing psychological well-being. The influence of comorbidity and the severity of depressive symptoms on the programme’s effectiveness will be explored through exploratory analyses. A mixed-methods approach is applied to relate older patients’ subjective experiences of change processes during the psychotherapy (as assessed by semi-structured interviews) to their psychometric treatment outcomes.

This large-scale superiority RCT is expected to provide robust evidence in support of the hypothesis that the intervention improves patient outcomes, thereby offering valuable guidance for the development of future healthcare policies. Consequently, the intervention is accompanied by a health economic evaluation. The hypotheses and questions to be examined in the study are presented in Tables 1 and 2.

**Table 1:** Overview of hypotheses in sub-study 1 (quantitative component of the mixed-methods design)

Hypothesis	Description
Main Hypothesis 1 H1a H1b	Our videoconference-based group-psychotherapy programme VISION-AGE (i.e., enriched LRT intervention group, IG) leads to greater reduction in • self-reported depression symptoms (primary outcome) and • clinician-rated depression symptoms (secondary outcome) compared to a videoconference-based non-clinical group discussion programme (i.e., active control group, CG) at post-treatment and 6-month FU, with moderate between and moderate-to-large within effect sizes for VISION-AGE, while we assume that both conditions lead to significant reduction in depression over time.
Hypothesis 2 H2a H2b	The VISION-AGE programme leads to • a greater reduction in anxiety symptoms and • a greater improvement in psychological well-being compared to the active control group at post-treatment and 6-month FU.
Hypothesis 3 H3a H3b H3c H3d H3e	The effectiveness of VISION-AGE is mediated by an improvement of self- and developmental regulation, as assessed in a standardized manner (i.e., • positive self-perceptions of ageing, • reminiscence, • life management, • future planning as mechanisms of action). The effect is most pronounced respectively in those participants who had lower baseline scores in these variables.

**Table 2:** Research questions in the mixed-methods design (quantitative and qualitative)

Research question	Description
Research question 1	Which subjectively experienced processes of change arise in the participants everyday life by participating in VISION-AGE?
Research question 2	How are the subjectively experienced processes of change related to the primary outcome?
Research question 3	Merging the results from study 1 and 2 (triangulation): How effective is VISION-AGE, and what are objective (measured via standardized instruments) and subjective (in the participants’ personal experience) mechanisms of action?
Research question 4	Explorative cost-effectiveness analysis: How cost-effective is VISION-AGE compared to the CG?

## Methods

This study protocol is reported in accordance with the SPIRIT (Standard Protocol Items: Recommendations for Interventional Trials [37] and the CONSORT (Consolidated Standards of Reporting Trials) statement [38] for transparent reporting of parallel-group randomised trials.

### Study design and timeline

**Fig. 1:**
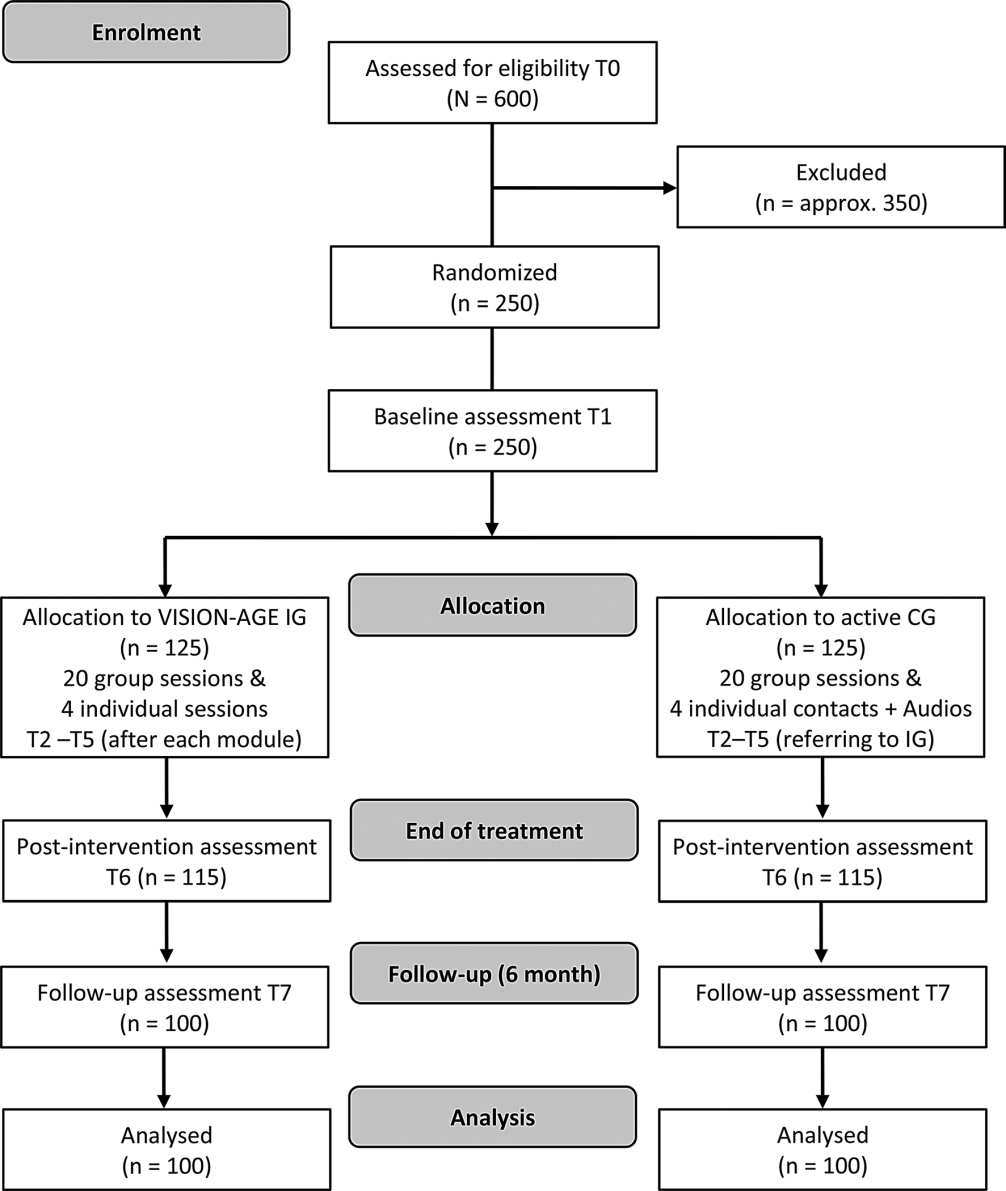
Flowchart of the VISION-AGE study design and participant allocation

The study is planned as a two-armed RCT with two groups, testing in Germany the superiority of the IG above a non-clinical, active CG. The IG includes therapist-guided psychotherapy, the CG comprises an active online discussion programme carried out by senior citizen assistants. A mixed-methods design, integrating both quantitative and qualitative data collection methodologies, will be employed. The study is planned to last 36 months, from 1 December 2025 to 30 November 2028, with recruitment continuing steadily until the target sample size is reached, overlapping with the intervention phase. As a fully online intervention, the trial enables nationwide recruitment across Germany. Participants can take part in the study from home using a suitable device. Figure 1 shows the trial procedure as a flow chart.

The study centre will be located at the Departments of Psychology at MSB Medical School Berlin and the University of Siegen. The group therapists and group facilitators can decide where to carry out digital interventions, as long as they are in a quiet space that ensures data security and privacy.

### Power and sample size

The study will aim to include 250 participants, with 125 allocated each to the IG and the CG, thereby ensuring a minimum of 100 completed data sets for each intervention at the FU. The plan is founded upon a priori sample size calculation, which yielded a total of 200 participants following a 1:1 randomisation ratio. However, the drop-out-rate must also be considered, which is estimated at 20%, based on the findings of previous studies, for example, 17% in a study with older adults experiencing depression and anxiety [36].

The sample size calculation is based on the assumption of a medium-to-large pre-post (“within”) effect size (d = 0.75) for VISION-AGE and a moderate “between” effect size (d = 0.45) for the contrast (pre-post change in the IG compared to pre-post change in the CG). It is hypothesised that the proposed programme, considered an enhanced form of LRT, will demonstrate effect sizes that are at least commensurate with those observed in LRT, and approximately equivalent to those reported for CBT: The large pre-post effect size, as found for CBT (d = 1.06) and LRT (d = 1.0) in late-life depression [16] will somewhat be reduced due to the possible comorbidity with anxiety and the video-based format (e.g., g = 0.79 for depression and g = 0.82 for anxiety, meta-analysis) [33]. The group setting additionally reduces the effect size in late-life depression (e.g., d = 0.65) [39]. However, since we are planning very small groups and additional individual sessions, we estimate a within effect size of no less than d = 0.75 and a between effect size of d = 0.45.

For statistical tests on the α = 5% level, with a total sample size of *N* = 200 (100 per arm), power simulations using R package Superpower [40] yielded a power > 0.90 for both main effect and the interaction regarding a 2 (condition) by 2 (time) mixed Analysis of variance (ANOVA), as well as power > 0.80 for the main contrast of interest (i.e., the pre-post change in the IG in comparison to the pre-post change in the CG).

### Recruitment of group therapists and group facilitators

It is planned to recruit geropsychologically qualified psychotherapists as study therapists via training institutions for psychotherapy with older adults. For the control group, certified senior citizen assistants (i.e., lower-qualified professionals supporting and caring for older adults in the community such as in senior citizens’ centres, parishes or at home) will be recruited.

### Recruitment of participants

A variety of strategies will be used to inform the target population about the study: In addition to leveraging the existing pool of prospective patients within the university’s psychotherapy outpatient clinics in Berlin and Siegen, a multifaceted approach involving self-referral and gatekeeper-referral strategies is planned. Specifically, it is intended to promote the study throughout Germany via articles in newspapers. We aim to inform health professionals as gatekeepers, including staff from geriatric and geropsychiatric hospitals and day clinics, general practitioners, as well as psychotherapists.

Prospective study participants can access information about the study VISION-AGE via the project website (www.vision-age.de). The study website provides key inclusion criteria to help interested individuals conduct an initial self-assessment of their eligibility and they can take a depression self-test using the Depression im Alter Skala (DIA-S) [41], without transmitting their data to the study centre. Individuals expressing interest in participating in the study are invited to contact the research team via telephone or a contact form on the study website. In the first step, their name and email address are collected exclusively for the purpose of study-related communication, such as the dissemination of study information and the first scheduling-appointment. The contact details are therefore entered into the study data capture system, which initiates the dispatch of an email containing instructions for study registration (including the provision of informed consent to participate in the study) on our primary data collection platform. The email also contains links to the study information and to available mental health crisis support services (each provided via a separate link, viewable without registration). Upon successful registration, participants receive an appointment for an enrolment interview, during which the study is presented and eligibility is assessed. In the absence of a response within a reasonable timeframe, the data is deleted.

### Inclusion criteria and accompanying measurement

The research team conducts enrolment interviews via videoconference with interested individuals to assess their suitability for the study. Therefore, at T0, measurement instruments that do not represent outcome variables are used to prevent selection bias based on the outcome measures themselves. Participants will be included in the study if they have the capacity to give informed consent, voluntarily agree to participate, and meet the following criteria: [a] they are aged 68+ (with no upper age limit); [b] they are diagnosed with mild or moderate unipolar depressive symptoms associated with one of the following ICD-10 diagnoses: F32.0/1, F33.0/1, F38.1/8, F41.2 or F43.2 based on a clinical interview; [c] they have clinically relevant depressive symptoms based on self-report; [d] they are not taking psychoactive medications or are taking a stable dosage for at least six weeks prior to study entry; [e] they have an indication for participation in group psychotherapy; [f] sufficient language and communication skills in German and [g] a functioning, stable internet connection and basic IT skills.

Participants diagnosed with severe depression are excluded from the study, as their condition necessitates more intensive care to ensure patient safety. Furthermore, the online format demands a substantial degree of autonomy, a quality that probably be hindered by severe depression. In addition, group-based interventions must take into account the potential burden on other participants that may arise from the intense self-disclosures of severely depressed participants.

Participants will be excluded from the study if: [a] they have very severe depressive symptoms based on the clinical interview (F32.2 or F33.2) or self-report; [b] they have very severe anxiety symptoms or symptoms of panic disorder or specific phobia that mandate in vivo exposure; [c] they report acute suicidal tendencies; [d] they show signs of, or have been diagnosed with dementia or mild cognitive impairment; [e] they report psychotic or bipolar symptoms or have been diagnosed with bipolar disorder or schizophrenia; [f] they have a physical illness in the terminal phase; or [g] they plan to change their intake of psychoactive medications before enrolment or during the study. Further reasons for exclusion include [h] receiving psychotherapy while participating in the study, [i] communication difficulties and visual or hearing impairments that would severely affect an individual’s ability to participate in the study. The reasons for excluding participants will be recorded.

During enrolment interviews, participants are asked to provide personal (contact) details such as age, address and phone number. In addition, information on pre-existing mental and physical comorbidities, current medication as well as relevant socio-demographic variables, is requested. Self-report measures of depressive and anxiety symptoms are collected via the Hospital Anxiety and Depression Scale (HADS) [42]. A HADS score of ≥ 8 is considered clinically relevant for the depression subscale, while a score of > 18 on either the depression or anxiety subscale indicates a very severe manifestation of that subscale. Depressive symptoms of clinical severity (i.e. a score of ≥ 4 on a 0–8 scale) and associated diagnoses are assessed using the section depression from the Diagnostisches Interview bei psychischen Störungen (Structured Clinical Interview for Mental Disorders, DIPS) [43], slightly adapted for the purpose of present study. Cognitive function is assessed using a slightly adapted version of the Mini Mental Status Test (MMST) [44], a screening tool for cognitive impairment. A score of MMST < 25 is considered to be (mild) cognitive impairment. An indication for participation in group therapy is assessed in the clinical impression, based on the assessment of whether the individual person with their symptoms, themes and personality could benefit from group exchange. Participants are assumed to have a stable internet connection and basic IT skills if they can register independently, provide digital informed consent and attend the enrolment interview.

Finally, the severity of the depressive disorder (mild or moderate) and the presence or absence of anxiety symptoms are assessed based on clinical judgement after all enrolment diagnostics have been carried out.

### Randomization and blinding

Participant eligibility is determined during the enrolment interview by a member of the research team, based on the inclusion and exclusion criteria. This information will be communicated to the participant and recorded in the data capture system. Following this evaluation, suitable participants are then randomly assigned to a study condition using a computer-generated simple randomisation algorithm. The research team has no influence on the allocation of suitable participants to one of the conditions. Within the specified conditions, patients are manually allocated to a block of four participants each, taking into account their time availability for group therapy. After the enrolment interview digital visits via videoconference will be carried out at baseline (T1) and post-treatment (T6) and FU (T7) by members of the research team who will be blinded to the group allocation and previous test results. The study data capture system implements a role-based access concept to ensure that details of group allocation and participants’ recorded data are only accessible to the Principal Investigator (PI) and research team members responsible for supervision.

### Intervention

Participants will find out in the first group session whether they have been assigned to the IG or the CG. Both programmes include 20 weekly online group appointments (with each session lasting 100 min, including a 10-min break) in a fixed group of four, as well as four additional individual meetings with group therapist (in the IG) or group facilitator (in the CG). Participants must join the meetings using their own devices, such as personal computers or tablets. If more than two participants permanently withdraw from a therapy group (e.g., due to illness or lack of motivation), the remaining participants will be offered the opportunity to switch to another group.

To prevent bias and buffer against potential investigator allegiance effects, group therapists and group facilitators are not part of the research team and do not conduct measurements with the participants. They are trained in a two-day pre-intervention workshop to ensure adherence to the respective manual and are regularly supervised by the research team based on session videos. In order to verify the integrity of the treatment, a sample of 10% of all sessions is evaluated by raters who are unaware of the study hypotheses, condition, and session number. Thereby adherence to the study protocol is evaluated using a 20-item adherence scale derived from the two manuals (10 Items IG, 10 Items CG). In addition, the session-documentation is regularly checked for manual-consistency.

### Intervention-group VISION-AGE

VISION-AGE is a standardised, age-sensitive group psychotherapy programme carried out via video conference by trained psychotherapists. The treatment manual was tested in the psychotherapeutic university outpatient clinic of the MSB Medical School Berlin during a three-year pilot phase and was refined in an iterative process based on clinical experiences and patient feedback. There was no evidence of any negative therapy effects. The manual focuses on promoting self- and developmental regulation strategies and thereby addresses the three biographical ‘time corridors’ past - present - future. Well-established LRT methods are enriched by CBT and PP techniques. In addition to the 20 group sessions, four individual sessions (each 45 min long) are intended to reinforce the therapeutic alliance and deepen the content of the group sessions. The initial upstream module focuses on reflecting upon and modifying existing images and experiences of ageing, as well as addressing ageism, in order to build the motivation necessary for change (Module 1 Positive self-perceptions of ageing), followed by Module 2 Life-review, Module 3 Life-management and Module 4 Life-planning. An accompanying handbook is sent to participants prior to the intervention, providing preparatory tasks related to specific sessions of the manual (e.g. collecting photos for the LRT sessions or a gratitude exercise for the Life-management sessions). For an overview of the therapeutic manual’s contents and the postulated mechanisms of action, see the supplemental material.

### Control group

The active CG is a non-clinical group discussion programme designed to provide social support and stimulation through group discussions in a supportive, non-judgemental environment. During the first half of each session, participants discuss personal, everyday issues from the previous week, followed by a discussion of stimulating, neutral topics such as hobbies, cooking, or animal companionship. Group facilitators encourage engagement and socialisation among group members, similar to the type of support participants might receive in a community support group at a senior citizens’ centre. The manual is based on one used in a previous study [36], where it showed a small positive effect on depression and anxiety in older patients, and has been adapted and expanded for the present study. The CG programme will be delivered by senior assistants, as the work requirements align with their usual work tasks and no clinical skills are necessary. To achieve the same therapeutic dosage as the IG, the time allocated to group therapy sessions is identical to that of the IG. In terms of individual contact, participants will receive four five-minute friendly digital meetings with their group moderator, as well as four additional 40-minute audio features offering stimulating content related to the preceding sessions (e.g., about the benefits of having an animal companion). See the supplemental material for an overview of the group discussion manual, including the discussion topics.

### Data acquisition, data management and data archiving

Participant data will be collected using a Primary Data Collection Tool based on MEDITyme^®^ platform technology as a web-based application, supplied by XPERTyme GmbH (Wessling, Germany). The platform has been developed to incorporate all the necessary features to enable secure video communications and to support the compliant collection, transmission, and storage of study data. It is designed to ensure compliance with Good Clinical Practice (GCP) and General Data Protection Regulation (GDPR) requirements. All source data and informed consent forms will be directly documented and stored on the platform, following predefined standardised processes. Since no additional source data are generated at the study sites, on-site monitoring is not required. In order to minimise errors in data entry and ensure data quality, the system incorporates predefined data entry rules and validation checks. Access to the platform is restricted to authorised study personnel and participants according to predefined roles and responsibilities and blinding specifications. For example, group therapists and group facilitators are only permitted to see the data of their own group participants. Any subsequent corrections are digitally documented and fully traceable through an audit trail. The group sessions and qualitative interviews will be recorded and subsequently stored in order to facilitate the supervision and qualitative analysis of the data.

The process evaluation will be conducted through continuous monitoring of data integrity, adherence to the informed consent procedure, and verification of protocol compliance by the Principal Investigator (PI), who has full access to all study data of all participants. Protocol deviations identified during monitoring will be documented and addressed according to CAPA (Corrective and Preventive Action) procedures.

In the primary data system, all members of the research team can view, within each participant’s history, whether the participant attended a group session (without any specific role assignment, only the session number, no content), a digital visit, or completed a questionnaire (attendance only, without access to the content). Thus, information on participant adherence is available without unblinding the research team. Due to the program’s regular group sessions, the group therapists and facilitators have a clear overview of participants’ adherence, which is discussed routinely during supervision meetings. The Primary Data Collection Tool enables automated reminder emails to be sent to participants, and in cases of dropout, telephone contact may be initiated to inquire about continued interest in participation.

Once the intervention phase is completed, following the last participant’s assessment, the technical support team by XPERTyme GmbH will generate two datasets from the study`s data capture before closing it: one with the source data and one with the relevant pseudonymized study data, suitable for statistical analysis. Both datasets will be protected with a checksum and archived separately from each other at the Medical School Berlin (MSB). Both the source data and the raw dataset will be retained for a minimum of 10 years in accordance with regulatory requirements. In addition, a member of the research team will generate a de-identified dataset from the pseudonymized data. After the retention period has expired, all records containing personal information will be securely deleted or destroyed, except for the de-identified dataset, as they no longer contain any personal reference. Access to identifiable information is strictly limited and may only be granted to auditors, or ethics committees for oversight purposes.

### Safety considerations

As part of the contact email sent to interested participants, a link is provided to a PDF containing information on always-available support services for crisis situations. In case of concerns or questions, participants may contact the research team at any time via email or by phone during designated office hours, or reach out to their group therapist or group facilitator during group or individual sessions, or through the messaging function within the Primary Data Collection Tool. Within the group sessions in both programmes, concepts for following supportive treatments are discussed, but such services are not directly provided or arranged for the participants.

The Primary Data Collection Tool facilitates the documentation of safety-relevant data that could potentially lead to an early termination of the study (see the section below for more information). An adverse event (AE) is defined as any undesirable experience that occurs to a participant during the trial and that may affect the intervention itself, the exclusion criteria or the participant’s ability to continue participating in the study. These events can be reported by participants or group therapists, group facilitators, or research team members at any time point during the study in the Data Collection Tool. Participants are regularly reminded of this option in the group sessions. Serious adverse events (SAEs), i.e. breach of trust in the study members, acute worsening of depressive symptoms, hospitalisation, self-harm, suicide attempts or suicide, will be documented in the case report form and reported to the main study site (MSB).

The study will also be monitored by an Independent Data and Safety Monitoring Board (IDSMB) specifically chosen to include a geriatrician, a geropsychiatric, a statistician and a clinical psychologist, ensuring the utmost competence and vigilance. The IDSMB will meet via videoconference before, during and after the trial to review its progress and provide an independent evaluation of the safety of the participating patients (especially in the event of SAEs) and adherence to the protocol (data integrity and the validity of the clinical trial). If necessary, the frequency of the web conferences can be increased. The study will only continue if it is deemed safe by the IDSMB, which can also provide the sponsor with information and make recommendations.

### Termination criterion

- If a participant withdraws their informed consent during the study, as termination criteria, all data collected up to that point will be removed from the analysis dataset, with the deletion duly documented.
  In addition, participation in the study may be terminated early under the following circumstances:
- If a participant does not wish to continue participating or cannot be reached, or fails to attend required study visits (drop-out);
- If a participant starts an ambulant psychotherapy between the assessment points T0 and T7;
- If a participant changes their psychoactive medication, continued study participation will be reviewed on a case-by-case basis in consultation with the participant and PI;
- If health problems arise that are not related to the study content, but may hinder focus-targeted or regular study participation, continued study participation will be reviewed on a case-by-case basis in consultation with the participant and PI. This includes new or worsening physical complaints (e.g. a fall followed by hospitalization), serious events (e.g. the unexpected death of a family member) or psychological complaints (e.g. acute grief – except for depression and anxiety, which are dealt with under point [f]);
- If any SAE occur, decisions regarding continued participation will always be made by the PI in consultation with the IDSMB, to ensure the participant’s well-being and the appropriateness of their involvement. The jointly made decision will be transparently communicated to the participant;
- If a participant passes away during the study period;
- If the PI or study sponsor decides to discontinue participation for specific reasons.

In the event of early termination, the termination criteria are recorded on an individual basis, specifying the corresponding time point and category.

### Assessment

Table 3 displays all quantitative measuring instruments together with their assessment time points over the course of the study.

In the selection of the measuring instruments, particular attention was paid to the objectivity, reliability, and validity of the assessments. The methods employed in this study encompass clinical interviews, questionnaires, cognitive tests and semi-structured qualitative interviews.

**Table 3:** Overview of descriptive data, quantitative outcomes and screening tools (with instruments and assessment time points)

Assessment Schedule	T0	T1	T2	T3	T4	T5	T6	T7
Informed Consent	x							
Allocation	x							
**Interventions**								
VISION-AGE IG								
CG								
**Inclusion/exclusion variables**								
• Personal contact data	x							
• Sociodemographic variables	x							
• Mental and physical comorbidities, medication	x							
• Depression and anxiety symptoms (HADS)	x							
• Modified Clinical interview for depression (based on DIPS)	x							
• Cognitive function (MMST)	x							
**Primary outcome**								
• Depression symptoms (PHQ-9)		x	x	x	x	x	x	x
**Secondary outcomes**								
• Clinician-Rated depression Symptoms (MADRS)		x					x	x
• Anxiety (GAS)		x					x	x
• Psychological Well-being (SPWB)		x					x	x
**Mediator variables**								
• Self-directed ageism (Subscale self-directed ageism, WHO Ageism Scale, expanded by four items)		x	x				x	x
• Ego-Integrity (NEIS)		x		x			x	x
• Selection Optimization Compensation (SOC)		x			x		x	x
• planning behaviour (based on the PFC)		x				x	x	x
**Further exploratory measures**								
• Working Alliance (WAI-SR)					x			
• Group Cohesion (FEPiG)					x			
• Programme Satisfaction (ZUF-8)							x	x
• Pre-programme: Assessment of individual therapeutic goals (across five categories); Post-programme: Goal attainment scale		x					x	x
• Assessment of medical resource utilization (self-developed questionnaire)		x					x	x
• Health-related life quality (EQ-5D-5L)		x					x	x
• Acceptance of video technology (TUQ)			x	x	x	x	x	x
• Assessment of health-related changes (self-developed questionnaire)		x	x	x	x	x	x	x
**Safety relevant data**	Notification is possible at anytime.							

Note. **T0** = Enrolment, afterward Allocation, **T1** = Baseline assessment, **T2–T5** = intermediate assessment, **T6** = post-treatment assessment, **T7** = FU assessment

The data collection will be conducted on seven time points to assess the short- and medium-term effects of the VISION-AGE-intervention, and will be identical for both the IG and CG. The assessment points include:

1. A baseline assessment prior to the start of the intervention;
2. Following completion of the first module (Positive self-perceptions of ageing, i.e., group session 4);
3. Following the second module (Life-review, i.e., group session 10);
4. Following the third module (Life-management, i.e., group session 15);
5. Following the fourth module (Life-planning, i.e., group session 19);
6. Two weeks after the end of the programme (i.e., group session 20); and
7. Six months post-programme (FU).

To encourage continued participation, the study participants receive greeting cards, which include memories of the group sessions, once they have completed all six assessment points.

### Primary outcomes

The primary outcome variable is the reduction in participants’ depressive symptoms, as indicated by changes in their self-reported scores on the Patient Health Questionnaire-9 (PHQ-9) [45] between pre- and post-treatment (T1–T6) and FU (T1–T7). The PHQ-9 is recorded at each measurement point T1–T7.

### Secondary outcomes

As secondary outcomes, changes from baseline (T1) measurement to posttreatment (T6) and FU (T7) will be examined in the following concepts:

- A reduction in clinician-rated depressive symptoms will be assessed using the Montgomery-Åsberg Depression Rating Scale (MADRS) [46];
- A reduction in anxiety symptoms will be assessed using the Geriatric Anxiety Scale (GAS) [47]; and
- An improvement in psychological well-being will be assessed using a short version the German short version of Ryff’s scales (SPWB) [48].

### Mediator variables

The following concepts of self- and developmental regulation strategies will be assessed as mediator variables at baseline (T1), at one of the four intermediate time points, after the completion of the respective module, at post-treatment (T6), and at FU (T7):

- Positive self-perceptions of ageing, fostered in Module 1, will be assessed as the inverse of the score on the self-directed ageism subscale of the World Health Organization (WHO) Ageism Scale (translated into German by the research team) [49] and is further expanded by four additional survey items on ageism [50];
- Reminiscence, fostered in Module 2, will be assessed as ego-integrity by using the short version of the Northwestern Ego Integrity Scale (NEIS; translated into German by the research team) [51];
- Life management, fostered in Module 3, will be assessed using the Scale Selection Optimisation Compensation (SOC) [52]; and
- Future planning, fostered in Module 4, will be assessed using a modified 6-item scale based on the Preparation for Future Care Needs – 5-item Short Scale (PFC; translated into German and modified by the research team) [53].

### Further exploratory measures

For exploratory purposes, following variables on specific group dynamics will be recorded at T4:

- The therapeutic alliance, as rated from the perspectives of both the patient and the group therapists or group facilitators, will be assessed using the Working Alliance Inventory-Short Revised (WAI-SR) [54] and
- Group Cohesion will be assessed using the Scale for the assessment of therapeutic processes in group therapy (FEPiG) [55].

Moreover, post-treatment (T6) and FU (T7), the general satisfaction of participants with the programme will be assessed using the Patient Satisfaction Questionnaire (ZUF-8) [56]. At baseline (T1), participants are supported in formulating SMART (specific, measurable, attractive, realistic and time-bound) individual therapy goals in five categories. At the post-treatment (T6) and FU (T7), participants will be asked to rate their progress towards these goals using a Goal Attainment Scale designed by the research team based on that of Wilz and colleagues. Additionally, participants’ assessment of medical resources utilization (health- and care-related treatments and assistive devices) will be assessed using a self-developed questionnaire at baseline, as well as at post-treatment and FU, so each measurement occurring approximately six months apart. At the same three points (T1, T6, T7) health-related life quality is assessed by using the EuroQol 5 Dimensions, 5 Levels Questionnaire (EQ-5D-5L) [57]. Acceptance of video technology is assessed at each measurement point (T2–T7) after the start of the programme by using the Telehealth Usability Questionnaire (TUQ) [58]. Changes in medication and new diagnoses made by participants’ outpatient physicians are inquired about at each assessment point.

### Planned statistical analyses

Participants who attend fewer than 75% of sessions (i.e. fewer than 15 group sessions and fewer than three individual sessions) will be excluded from the per-protocol analysis. To prevent an attrition bias, intention-to-treat analyses are used. Missing data will be handled using multiple imputation.

### Statistical analysis sub-study 1 (quantitative study)

Statistical analyses regarding sub-study 1 will be based on intention-to-treat (ITT) as well as on per-protocol analyses. The primary analysis will be ITT. To compensate for panel attrition and item-nonresponse, missing data will be multiply imputed in all ITT analyses. Imputation models will be fully compatible to the subsequent analysis models (i.e., substantive model compatible multiple imputation) and will include all relevant associations (like interaction terms) and will represent the clustered structure of the data. The main substantive model of interest is a (generalized) linear mixed effects model, which evaluates stability and change in the outcome variables over time depending on experimental condition. The focal effect of interest in this model is the time by condition interaction. To interpret this effect, we especially look at the following contrast: the pre to post change in the experimental condition in comparison to the pre to post change in the control condition (i.e., a contrast over two contrasts). Additionally, pre-post and pre-FU effect sizes (Cohen’s d) will be calculated for all outcome-variables for both groups (IG and CG) to further quantify the found effects. In additional (exploratory) analyses, we will test for possible effects of gender, age, education, comorbidity and severity of comorbid symptoms. We also explore possible interactions of the outcome variables by means of parallel longitudinal path modelling. If the first two hypotheses can be confirmed, hypothesis 3 (regarding the underlying therapeutic mechanisms) will be evaluated by mediation and moderation analyses using path models.

### Statistical analysis sub-study 2 (qualitative study)

The question of sub-study 2 is which subjective processes of change the IG participants experience in everyday life as a result of participating in VISION-AGE (step 1; qualitative approach), and how these changes are associated with quantitative therapy outcomes (step 2; quantitative approach).

In step 1, a selection of IG participants are asked about their subjective experiences in structured, qualitative interviews. In accordance with the principle of maximum variation within the sample, the IG participants who are interviewed will have achieved different outcomes in the post-treatment (i.e. participants who improved as well as those who deteriorated). We estimate that roughly 20 IG participants will be needed to reach theoretical saturation. The interviews will be recorded on video and fully transcribed. Through qualitative content analysis categories will be developed based on the written material. These categories will represent different types of subjective processes of change, such as ‘rediscovering resources’ and ‘defining life goals’. Based on these categories, shorter, more focused, problem-centered interviews will be conducted with all remaining IG participants (approximately 80 at the post- treatment stage, considering drop-outs), asking specifically about the identified categories.

In step 2, it is recorded for each IG participant whether various categories of subjective change processes apply to them (yes/no). Additionally, an individual frequency score (i.e. how often each category is used) and an individual diversity score (i.e. the number of categories used at least once) are calculated for each IG participant [35].

### Triangulation of sub-study 1 and 2

To gain a deeper understanding, findings from sub-studies 1 and 2 are triangulated by relating the categories of subjective change processes identified in the qualitative analysis of sub-study 2 to the mediators identified in sub-study 1 [35]. Finally, those subjective and objective processes of change that have been proven as the best predictors in the respective sub-study (i.e., 1 and 2) are statistically put in relation in terms of their predictive power in a common regression model.

### Cost-effectiveness

An analysis of economic gains will be conducted by calculating the incremental cost-effectiveness ratio, defined as the difference in total costs divided by the difference in effect between the IG and the CG. The effect will be operationalized in terms of quality-adjusted life years (QALYs). To assess QALYs, the EQ-5D-5L [57] will be used, covering five dimensions—mobility, self-care, usual activities, pain/discomfort, and anxiety/depression—each with five levels of functioning: no problems, slight problems, moderate problems, severe problems, and extreme problems/unable to.

## Discussion

VISION-AGE is a large, nationwide superiority RCT to investigate the effectiveness and mechanisms of action of online group psychotherapy for older adults with clinically significant depression symptoms and partially comorbid anxiety in Germany. This approach is intended to address existing research gaps while facilitating a systematic analysis of both quantitative and qualitative developments throughout the programme’s duration. This systematic analysis is underpinned by the implementation of a mixed-methods design. The VISION-AGE programme builds on evidence-based therapeutic strategies from LRT, incorporating additional features proven to be helpful in self- and developmental regulation, as well as techniques from CBT and PP.

The therapeutic concept of the VISION-AGE manual is based on the strengthening of four self-regulatory resources, which will be examined as mediator variables. In the first step, participation in the program is expected to foster a more positive attitude toward one’s own ageing while reducing self-directed ageism. In the second step, the intervention aims to facilitate a balanced review of the patient’s life. In the third step, the training of SOC-strategies is intended to enhance life-management skills. In the fourth step, within the framework of the manual, participants are encouraged to actively engage with future-related issues and potential anxieties, thereby strengthening their capacity to plan for upcoming challenges. In this way, the ‘time corridors’ past - present - future are intensively discussed, whereby the latter has received little attention in previous therapeutic approaches. The group component is expected to further promote the ability to build relationships, develop trust, and strengthen both generational identity (based on being born in the same historical period) and age identity (based on sharing a similar life stage). These factors have a direct mood-enhancing impact, and thereby potentially improve mental and physical health.

It is hypothesised that VISION-AGE is more effective than an active control group, designed to resemble a typical social group in settings such as social care, at reducing symptoms of depression. The study examines whether strengthening the four mediating strategies is a decisive developmental step in alleviating depression and anxiety symptoms and enhancing participants’ well-being. Additionally, qualitative interviews provide insight into the change processes from the participants’ perspectives. These can provide new insights into important concepts that may not have been previously considered.

Together, the age-adapted structure of the manual and the comprehensive, multidimensional outcome assessment (both quantitative and qualitative) reflect the programme’s broad scope and its potential to promote holistic, active, and positive living in later life. The study results will provide insights into whether older adults accept online group therapy and to what extent they benefit from it.

Despite its promising theoretical foundation and practical relevance, the VISION-AGE study has some potential limitations. First, a selection bias may occur because participation requires basic IT skills; as a result, younger (under 80), more educated, or more motivated individuals may be overrepresented, which could limit the generalisability of the findings. Second, while the small group size is intended to foster intimacy and openness, it also increases the likelihood that individual participants will have a disproportionately strong influence on the perceived group cohesion and overall group dynamics. Third, although we believe that the economic, social, and ethical advantages of online group therapy outweigh its disadvantages, this specific delivery format might underestimate the effect of the VISION-AGE programme. Fourth, the intervention targets older adults with depressive symptoms, with or without comorbid anxiety, which may limit the generalisability of the findings to younger cohorts or older adults with different disorders.

### Conclusion

The VISION-AGE manual is a pioneering treatment program that combines innovative elements to deliver a specialized transdiagnostic clinical care intervention for the underserved populations of older adults. As an online group therapy, it increases access to psychotherapy and is easy to implement regardless of patients’ location, mobility, or tight time schedules. The program aims to reduce depression and anxiety symptoms while enhancing psychological well-being and self- and developmental regulation strategies. Including a cost-effectiveness analysis, the study provides practical insights to support evidence-based healthcare planning and guide broader implementation across care settings.

In the event of a positive programme effect, the clinical effectiveness of VISION-AGE should be investigated under diverse conditions regarding various mental health disorders (e.g., prolonged grief, singular anxiety disorder), more vulnerable older populations (e.g., patients with high levels of multimorbidity and/or cognitive impairment) and considering other formats (i.e., face-to-face treatment; individual therapy) and therapeutic dosages (e.g., fewer sessions). To counteract potential investigator allegiance effects, future evaluations of the VISION-AGE manual should be conducted by independent external (preferably international) research teams. The goal is to implement an efficient, cost-effective transdiagnostic psychotherapy concept that is accessible and appealing to older patients, and thereby improving mental health care for this underserved population.

### Trial status

The study is scheduled to begin on 1 December 2025, and conclude on 30 November 2028.

## Supplementary Information

Below is the link to the electronic supplementary material.

Supplementary Material 1

Supplementary Material 2

## Data Availability

No datasets were generated or analysed during the current study.

## Declarations

### Protocol amendments

Important protocol modifications will be reported in the trial paper.

### Roles and responsibilities

- *Name of trial sponsor*: MSB Medical School Berlin, Department of Psychology, Rüdesheimer Str. 50, 14197 Berlin, Germany(PI); University of Siegen (Co-PI), Department of Psychology, Adolf-Reichwein-Str. 2a, 57068 Siegen, Germany (Co-PI).
- *Supporting participating groups/bodies*: Center for Clinical Studies Cologne (CTC Cologne): Professional advice regarding: (1) Legal requirements and ICH/GCP (2) Creation of a data protection concept. Programming of the study software MEDITyme^®^ as a white-label solution by XPERTyme GmbH. Members of the IDSMB: PD Dr. Steffen Unkel, Biostatistician, Institute for Medical Statistics, Göttingen University Medical Centre, Germany. Prof. Dr. Heide Glaesmer, Psychologist, Department of Medical Psychology and Medical Sociology, Leipzig University Hospital, Germany. Prof. Dr. Michael Hüll, Geropsychiatrist, Emmendingen Center for Psychiatry, Clinic for Geriatric Psychiatry and Psychotherapy, Germany. Prof. Helmut Frohnhofen, Geriatrician, University Hospital Düsseldorf, Department of Geriatric Medicine, Germany.

### Access to data

All regulatory requirements relating to data protection will be met. Subsequent to the completion and publication of all planned publications, the trials’ de-identified data, accompanied by a data dictionary, will be stored and made accessible via the Research Data Centre of the Leibniz Institute for Psychology.

### Dissemination policy

In line with DFG form 2.00–1/21, we will publish our project results for the purpose of scholarly communication in open access (directly in international, high-impact open access journals or open access platforms, or in addition to commercial publication they will be included in subject-specific or institutional electronic repositories). Results will be published in accordance with CONSORT statement for reporting of randomized controlled trials (sub-study 1) and JARS-Qual (sub-study 2). In addition, a workshop for psychotherapist’s training will be designed and given at psychotherapy training events in order to disseminate the treatment concept in the field. A senior citizen organization will be asked to summarize the findings in user-friendly language and ensure that the information is accessible to a public audience and the target population.

## Abbreviations

AE: Adverse event
ANOVA: Analysis of variance
CAPA: Corrective and Preventive Action
CBT: Cognitive and behavioural therapy
CG: Control Group
DFG: German Research Foundation [Deutsche Forschungsgemeinschaft]
DIPS: Diagnostisches Interview bei psychischen Störungen [Diagnostic Interview for Psychological Diseases]
EQ-5D-5L: EuroQol 5 Dimensions, 5 Levels Questionnaire
FU: Follow-up
GAS: Geriatric Anxiety Scale
GCP: Good clinical practice
FEPiG: Scalefor the assessment of therapeutic processes in group therapy
GDPR: General Data Protection Regulation
HADS: Hospital Anxiety and Depression Scale
IDSMB: Independent Data and Safety Monitoring Board
IG: Intervention Group
LRT: Life review therapy
MADRS: Montgomery-Åsberg Depression Rating Scale
MMST: Mini Mental Status Test
MSB: Medical School Berlin
NEIS: Northwestern Ego Integrity Scale – Short
PFC: Preparation for Future Care Needs – Short
PHQ-9: Patient Health Questionnaire-9
PI: Principal investigator
PP: Positive psychology
QALY: Quality-adjusted life years
RCT: Randomized controlled trial
Ryff-Scale: Scales of psychological well-being
SAE: Serious adverse event
SOC: selecting, optimising and compensating
SPWB: German short version of Ryff’s scales of psychological well-being
TUQ: Telehealth Usability Questionnaire
WAI-SR: Working Alliance Inventory- Short Revised
WHO: World Health Organization
ZUF-8: Patient Satisfaction Questionnaire

## References

[1] Andreas S, Schulz H, Volkert J, Dehoust M, Sehner S, Suling A, et al. Prevalence of mental disorders in elderly people: the European MentDis_ICF65 + study. Br J Psychiatry. 2017;210:125–31. 10.1192/bjp.bp.115.180463. 27609811 10.1192/bjp.bp.115.180463

[2] Zenebe Y, Akele B, W/Selassie M, Necho M. Prevalence and determinants of depression among old age: a systematic review and meta-analysis. Ann Gen Psychiatry. 2021;20:55. 10.1186/s12991-021-00375-x. 34922595 10.1186/s12991-021-00375-x PMC8684627

[3] Alexopoulos GS. Mechanisms and treatment of Late-Life depression. Focus. 2021;19:340–54. 10.1176/appi.focus.19304. 34690604 10.1176/appi.focus.19304 PMC8475935

[4] Miller R, Chin S, Sedai AK. The welfare cost of late-life depression. J Econ Behav Organ. 2022;204:15–36. 10.1016/j.jebo.2022.10.001.

[5] Canuto A, Weber K, Baertschi M, Andreas S, Volkert J, Dehoust MC, et al. Anxiety disorders in old age: psychiatric Comorbidities, quality of Life, and prevalence according to Age, Gender, and country. Am J Geriatr Psychiatry. 2018;26:174–85. 10.1016/j.jagp.2017.08.015. 29031568 10.1016/j.jagp.2017.08.015

[6] Kryza-Lacombe M, Kassel MT, Insel PS, Rhodes E, Bickford D, Burns E, et al. Anxiety in late-life depression is associated with poorer performance across multiple cognitive domains. J Int Neuropsychol Soc. 2024;30:807–11. 10.1017/S1355617724000262. 39291416 10.1017/S1355617724000262 PMC11735324

[7] King-Kallimanis B, Gum AM, Kohn R. Comorbidity of depressive and anxiety disorders for older Americans in the National comorbidity Survey-Replication. Am J Geriatr Psychiatry. 2009;17:782–92. 10.1097/JGP.0b013e3181ad4d17. 19700950 10.1097/JGP.0b013e3181ad4d17

[8] Frost R, Nair P, Aw S, Gould RL, Kharicha K, Buszewicz M, et al. Supporting frail older people with depression and anxiety: a qualitative study. Aging Ment Health. 2020;24:1977–84. 10.1080/13607863.2019.1647132. 31619050 10.1080/13607863.2019.1647132 PMC8842711

[9] Forstmeier S, Zimmermann S, Van Der Hal E, Auerbach M, Kleinke K, Maercker A, et al. Effect of life review therapy for holocaust survivors: A randomized controlled trial. J Trauma Stress. 2023;36:628–41. 10.1002/jts.22933. 37155933 10.1002/jts.22933

[10] Forstmeier S, Maercker A, Bohli L, Savaskan E, Roth T. Cognitive behavioural treatment for mild alzheimer’s patients and their caregivers (CBTAC): results of a randomised controlled trial. Aging Ment Health. 2025;29:359–68. 10.1080/13607863.2024.2393748. 39164933 10.1080/13607863.2024.2393748

[11] Hesse M, Forstmeier S, Cuhls H, Radbruch L. Volunteers in a biography project with palliative care patients – a feasibility study. BMC Palliat Care. 2019;18:79. 10.1186/s12904-019-0463-0. 31590633 10.1186/s12904-019-0463-0 PMC6781359

[12] Coupland C, Dhiman P, Morriss R, Arthur A, Barton G, Hippisley-Cox J. Antidepressant use and risk of adverse outcomes in older people: population based cohort study. BMJ. 2011;343(02 1):d4551–4551. 10.1136/bmj.d4551. 21810886 10.1136/bmj.d4551 PMC3149102

[13] Demmerle C, Gellert P, Kessler E-M. Psychotherapists’ experiences providing at-home psychotherapy for home-living older adults with long-term care needs and depression. J Couns Psychol. 2023;70:403–14. 10.1037/cou0000663. 37036680 10.1037/cou0000663

[14] Cuijpers P, Karyotaki E, Eckshtain D, Ng MY, Corteselli KA, Noma H, et al. Psychotherapy for depression across different age groups: A systematic review and Meta-analysis. JAMA Psychiatry. 2020;77:694. 10.1001/jamapsychiatry.2020.0164. 32186668 10.1001/jamapsychiatry.2020.0164 PMC7081149

[15] Huang AX, Delucchi K, Dunn LB, Nelson JC. A systematic review and Meta-analysis of psychotherapy for Late-Life depression. Am J Geriatr Psychiatry. 2015;23:261–73. 10.1016/j.jagp.2014.04.003. 24856580 10.1016/j.jagp.2014.04.003

[16] Pinquart M, Duberstein PR, Lyness JM. Effects of psychotherapy and other behavioral interventions on clinically depressed older adults: A meta-analysis. Aging Ment Health. 2007;11:645–57. 10.1080/13607860701529635. 18074252 10.1080/13607860701529635

[17] Pinquart M, Forstmeier S. Effects of reminiscence interventions on psychosocial outcomes: A meta-analysis. Aging Ment Health. 2012;16:541–58. 10.1080/13607863.2011.651434. 22304736 10.1080/13607863.2011.651434

[18] Raue PJ, McGovern AR, Kiosses DN, Sirey JA. Advances in psychotherapy for depressed older adults. Curr Psychiatry Rep. 2017;19:57. 10.1007/s11920-017-0812-8. 28726061 10.1007/s11920-017-0812-8 PMC6149527

[19] Gould RL, Coulson MC, Howard RJ. Cognitive behavioral therapy for depression in older people: A Meta-Analysis and Meta‐Regression of randomized controlled trials. J Am Geriatr Soc. 2012;60:1817–30. 10.1111/j.1532-5415.2012.04166.x. 23003115 10.1111/j.1532-5415.2012.04166.x

[20] Dafsari FS, Bewernick B, Böhringer S, Domschke K, Elsaesser M, Löbner M, et al. Cognitive behavioral therapy for Late-Life depression (CBTlate): results of a Multicenter, Randomized, Observer-Blinded, controlled trial. Psychother Psychosom. 2023;92:180–92. 10.1159/000529445. 37004508 10.1159/000529445

[21] Wuthrich VM, Meuldijk D, Jagiello T, Robles AG, Jones MP, Cuijpers P. Efficacy and effectiveness of psychological interventions on co-occurring mood and anxiety disorders in older adults: A systematic review and meta‐analysis. Int J Geriatr Psychiatry. 2021;36:858–72. 10.1002/gps.5486. 33368598 10.1002/gps.5486

[22] Sittler MC, Lechner-Meichsner F, Wilz G, Kessler E. Does age matter? Initial treatment goals of older adults with major depression in outpatient cognitive behavioural therapy. Clin Psychol Psychother. 2022;29:554–66. 10.1002/cpp.2646. 34254717 10.1002/cpp.2646

[23] Forstmeier S, Maercker A, editors. Der Lebensrückblick in therapie und beratung: Ansätze der Biografiearbeit, reminiszenz und Lebensrückblicktherapie. Berlin, Heidelberg: Springer Berlin Heidelberg; 2024. 10.1007/978-3-662-68077-3.

[24] Cappeliez P, O’Rourke N. Empirical validation of a model of reminiscence and health in later life. J Gerontol B Psychol Sci Soc Sci. 2006;61:P237–44. 10.1093/geronb/61.4.P237. 16855036 10.1093/geronb/61.4.p237

[25] Hu RX, Luo M, Zhang A, Li LW. Associations of ageism and health: A systematic review of quantitative observational studies. Res Aging. 2021;43:311–22. 10.1177/0164027520980130. 33317402 10.1177/0164027520980130

[26] Freund AM, Baltes PB. Toward a theory of successful aging: Selection, optimization, and compensation. In: FerFernández-Ballesteros R, editor. Geropsychology: European perspectives for an aging world. Göttingen, Germany: Hogrefe & Huber; 2007. pp. 239–54.

[27] Sörensen S, Mak W, Chapman B, Duberstein PR, Lyness JM. The relationship of Preparation for future care to depression and anxiety in older primary care patients at 2-Year Follow-up. Am J Geriatr Psychiatry. 2012;20:887–94. 10.1097/JGP.0b013e31822ccd8c. 21952122 10.1097/JGP.0b013e31822ccd8c PMC3458161

[28] Tavares LR, Barbosa MR. Efficacy of group psychotherapy for geriatric depression: A systematic review. Arch Gerontol Geriatr. 2018;78:71–80. 10.1016/j.archger.2018.06.001. 29933137 10.1016/j.archger.2018.06.001

[29] Montepare JM. Subjective age: toward a guiding lifespan framework. Int J Behav Dev. 2009;33:42–6. 10.1177/0165025408095551.

[30] Kessler E-M. Psychotherapeutisches arbeiten Mit Alten und Sehr Alten menschen. Stuttgart, Germany: Kohlhammer; 2021.

[31] Snarr JD, Slep AMS, Grande VP. Validation of a new self-report measure of parental attributions. Psychol Assess. 2009;21:390–401. 10.1037/a0016331. 19719350 10.1037/a0016331

[32] Goodarzi Z, Holroyd-Leduc J, Seitz D, Ismail Z, Kirkham J, Wu P, et al. Efficacy of virtual interventions for reducing symptoms of depression in community-dwelling older adults: a systematic review. Int Psychogeriatr. 2023;35:131–41. 10.1017/S1041610222000412. 35603891 10.1017/S1041610222000412

[33] Păsărelu CR, Andersson G, Bergman Nordgren L, Dobrean A. Internet-delivered transdiagnostic and tailored cognitive behavioral therapy for anxiety and depression: a systematic review and meta-analysis of randomized controlled trials. Cogn Behav Ther. 2017;46:1–28. 10.1080/16506073.2016.1231219. 27712544 10.1080/16506073.2016.1231219

[34] Castonguay LG, Barkham M, Lutz W, McAleavey AA, editors. Bergin and Garfield’s handbook of psychotherapy and behavior change. 7th ed. Hoboken (NJ): John Wiley & Sons; 2013. p. 583–624.

[35] Wucherpfennig F, Boyle K, Rubel JA, Weinmann-Lutz B, Lutz W. What sticks? Patients’ perspectives on treatment three years after psychotherapy: A mixed-methods approach. Psychother Res. 2020;30:739–52. 10.1080/10503307.2019.1671630. 31559926 10.1080/10503307.2019.1671630

[36] Wuthrich VM, Rapee RM, Kangas M, Perini S. Randomized controlled trial of group cognitive behavioral therapy compared to a discussion group for co-morbid anxiety and depression in older adults. Psychol Med. 2016;46:785–95. 10.1017/S0033291715002251. 26498268 10.1017/S0033291715002251

[37] Chan A-W, Tetzlaff JM, Altman DG, Laupacis A, Gøtzsche PC, Krleža-Jerić K, et al. SPIRIT 2013 statement: defining standard protocol items for clinical trials. Ann Intern Med. 2013;158:200–7. 10.7326/0003-4819-158-3-201302050-00583. 23295957 10.7326/0003-4819-158-3-201302050-00583 PMC5114123

[38] Schulz KF, Altman DG, Moher D, for the CONSORT Group. CONSORT 2010 statement: updated guidelines for reporting parallel group randomised trials. BMJ. 2010;340:c332–332. 10.1136/bmj.c332. mar23 1. 20332509 10.1136/bmj.c332 PMC2844940

[39] Spek V, Nyklíček I, Smits N, Cuijpers P, Riper H, Keyzer J, et al. Internet-based cognitive behavioural therapy for subthreshold depression in people over 50 years old: a randomized controlled clinical trial. Psychol Med. 2007;37:1797–806. 10.1017/S0033291707000542. 17466110 10.1017/S0033291707000542

[40] Lakens D, Caldwell AR. Simulation-Based power analysis for factorial analysis of variance designs. Adv Methods Pract Psychol Sci. 2021;4:2515245920951503. 10.1177/2515245920951503.

[41] Heidenblut S, Zank S. Entwicklung eines Neuen depressionsscreenings für Den einsatz in der geriatrie: die „Depression-im-Alter-Skala (DIA-S). Z Für Gerontol Geriatr. 2010;43:170–6. 10.1007/s00391-009-0067-z. 10.1007/s00391-009-0067-z 19760357

[42] Herrmann-Lingen C, Buss U, Snaith RP. HADS-D hospital anxiety and depression Scale - Deutsche Version. 4., aktualisierte und Neu Normierte auflage. Göttingen, Germany: Hogrefe; 2018.

[43] Margraf J, Cwik JC, Von Brachel R, Suppiger A, Schneider S. DIPS open access 1.2: diagnostisches interview Bei Psychischen Störungen. Ruhr-Universität Bochum (RUB); 2021. 10.46586/rub.172.149.

[44] Folstein MF, Folstein SE, McHugh PR. Mini-mental state. J Psychiatr Res. 1975;12:189–98. 10.1016/0022-3956(75)90026-6. 1202204 10.1016/0022-3956(75)90026-6

[45] Kliem S, Sachser C, Lohmann A, Baier D, Brähler E, Gündel H, et al. Psychometric evaluation and community norms of the PHQ-9, based on a representative German sample. Front Psychiatry. 2024;15:1483782. 10.3389/fpsyt.2024.1483782. 39726913 10.3389/fpsyt.2024.1483782 PMC11670475

[46] Schmidtke A, Fleckenstein P, Moises W, Beckmann H. Studies of the reliability and validity of the German version of the Montgomery-Asberg depression rating scale (MADRS). Schweiz Arch Neurol Psychiatr Zurich Switz 1985. 1988;139:51–65. 2455937

[47] Gottschling J, Segal DL, Häusele C, Spinath FM, Stoll G. Assessment of anxiety in older adults: translation and psychometric evaluation of the German version of the geriatric anxiety scale (GAS). J Psychopathol Behav Assess. 2016;38:136–48. 10.1007/s10862-015-9504-z.

[48] Tibubos AN, Reinwarth AC, Reiner I, Werner AM, Wild PS, Münzel T, et al. Psychological indicators for healthy aging: validation of the German short version of ryff’s scales of psychological well-being (SPWB). J Patient-Rep Outcomes. 2025;9:25. 10.1186/s41687-025-00854-9. 39998720 10.1186/s41687-025-00854-9 PMC11861829

[49] Murray AL, De La Fuente-Núñez V. Development of the item pool for the ‘WHO-ageism scale’: conceptualisation, item generation and content validity assessment. Age Ageing. 2023;52(Suppl_4):iv149–57. 10.1093/ageing/afad105. 10.1093/ageing/afad105 PMC10615060 37902522

[50] Kessler E-M, Warner L-M. Ageismus – Altersbilder und altersdiskriminierung in Deutschland [Ageism – Images of aging and age discrimination in Germany]. Report. Berlin: Federal Anti-Discrimination Agency; 2023.

[51] Janis L, Canak T, Machado MA, Green RM, McAdams DP. Development andvalidation of the northwestern Ego integrity scale. Nortwestern University. 2011

[52] Baltes PB, Baltes MM, Freund AM, Lang F. The measurement of selection, optimization, and compensation (SOC) by self report. 1999. Available from: 10.13140/RG.2.1.2213.4807.

[53] Sörensen S, Chapman BP, Duberstein PR, Pinquart M, Lyness JM. Assessing future care Preparation in late life: two short measures. Psychol Assess. 2017;29:1480–95. 10.1037/pas0000446. 28301191 10.1037/pas0000446 PMC5600625

[54] Wilmers F, Munder T, Leonhart R, Herzog T, Plassmann R, Barth J, et al. Die deutschsprachige version des working alliance Inventory - short revised (WAI-SR) - Ein schulenübergreifendes, ökonomisches und empirisch validiertes instrument Zur erfassung der therapeutischen allianz. Klin Diagn Eval. 2008;1:343–58.

[55] Vogel E, Blanck P, Bents H, Mander J. Wirkfaktoren in der Gruppentherapie: Entwicklung und Validierung eines Fragebogens [Change factors in group therapy: Development and validation of a questionnaire]. PPmP Psychother Psychosom Med Psychol. 2016;66(5):170–79. 10.1055/s-0042-04495FEPiGFEPiGFEPiGFEPiG. 10.1055/s-0042-104495 27128826

[56] Schmidt J, Lamprecht F, Wittmann WW. Satisfaction with inpatient management. Development of a questionnaire and initial validity studies. Psychother Psychosom Med Psychol. 1989;39:248–55. 2762479

[57] Herdman M, Gudex C, Lloyd A, Janssen Mf, Kind P, Parkin D, et al. Development and preliminary testing of the new five-level version of EQ-5D (EQ-5D-5L). Qual Life Res. 2011;20:1727–36. 10.1007/s11136-011-9903-x. 21479777 10.1007/s11136-011-9903-x PMC3220807

[58] Zimmermann J, Morf H, Scharf F, Knitza J, Moeller H, Muehlensiepen F, et al. German version of the telehealth usability questionnaire and derived short questionnaires for usability and perceived usefulness in health care assessment in telehealth and digital therapeutics: instrument validation study. JMIR Hum Factors. 2024;11:e57771. 10.2196/57771. 39571151 10.2196/57771 PMC11621722

