# Supplementary material for: Age-sensitive telehealth group therapy for depression in older adults with and without comorbid anxiety (VISION-AGE): study protocol for a randomized controlled trial in an embedded mixed-methods design
Source: BMC Psychiatry. 2026 Feb 18;26:215. doi: 10.1186/s12888-026-07851-2 (PMC12937509; doi:10.1186/s12888-026-07851-2)
Supplement: Supplementary file 1 — Supplementary Material 1 [file 12888_2026_7851_MOESM1_ESM.docx]

# Supplemental material

VISION-AGE Therapy session overview © Prof. Dr. Eva-Marie Kessler & Prof. Dr. Simon Forstmeier 2021

| Session Nr. | Module Focus/ Content of Session | Focused Mode of Action |
| --- | --- | --- |
| 1 | **Introduction, familiarisation** |  |
| 2-4 | **Reflexion and modification of negative attitudes towards ageing**  ***Guided exploration and disputation of self-perceptions of ageing***  i.e., comparison of positive vs. negative aspects of getting older/ageing (“What are positive/negative experiences you have made in regard to getting older?”) | Detecting own age stereotypes and promoting a differentiated, potential-oriented self-perceptions of ageing, aiming to enable participants to perceive positive aspects and potential for change in old age “despite” being old |
| 5-10 | **Life-review (“Looking back “)**  ***Resource-oriented Life-review Intervention***  One session each (session 5, 6, 7, 8, 9) on:   1. lifeline 2. Childhood, origin family etc. 3. Emerging adulthood 4. achievements, resources 5. challenges and how to overcome them   Stimulating the remembrance of positive memories and incitement of re-evaluation of negative biographic experiences  Use of biographically significant materials that participants bring in  Session 10: Review session (i.e., “What things or experiences in your life have made you into the person you have become?” “What are values and guiding principles you obtained? “ | Promotion of adaptive life-review strategies with the aim of becoming aware of one’s own strengths as well as a differentiated, more meaningful evaluation and reconstruction of one’s own biography (ego integrity) |
| 11-15 | **Life-management (“Looking at the here and now “)**  ***Focusing on and building of every-day resources***  Session 11: Deepening Resources by directing attention to subjective  a) Strengths of character/skills  b) social relations  c) material goods  d) Places/Services  (including use of working materials based on positive psychology: VIA character strengths; Circle of Support; Resource cards)  Session 12: “What makes a day a good day? What did I do/feel/experience/think on a good day?” (Gratitude / Positive diary)  Session 13: Stress skills (dealing with restrictions, reducing demands, concentrating on the essentials)  Session 14: Shaping social relationships (forming social skills, i.e. saying no, asking for help)  Session 15: Integration of previous lessons for a positive lifestyle | Promotion of a “positivity  orientation“ in the sense of selecting strengths, resources and subjectively significant people and activities, optimization and compensation with the aim of improving self-esteem, internal control and positive routines |
| 16-19 | **Life-planning (“Looking ahead “)**  ***Resource-oriented future planning***  Confronting expectations towards one’s own future, including positive and negative future-scenarios (goals, hopes, dreams, fears, concerns) Collection/visualization Four future-domains (1 session per domain):   - Housing situation, forms of housing (session 16) - Health, care, health care proxy (session 17) - Death, burial, inheritance, living will (session 18) - Plans and wishes (session 19)   For each of the four sessions, participants should become aware of their limits and potentials of exerting influence (omnipotence – powerlessness – “partial power”, based on the Circle of Influence)   - “What are aspects you cannot control (but can try accepting)?” - “What can you directly influence, what can you shape or help shape?” - “Out of the things you can control, what could you possibly do next?” | Promoting the disputation of feared future scenarios and adaptive strategies of future planning in the area of conflict between control and acceptance |
| 20 | **Reviewing the therapy**  Each participant names a symbol/metaphor for their “new” image of ageing (image of impulses from the Zurich resource model)  **Group Farewell** | Diachronic integration of the four modules |

VISION-AGE Control group session overview

The control group manual was adapted and expanded based on the following manual:

Wuthrich V, Kangas M. Socialisation, Stimulation & Support Group Program: Training Manual. Sydney: Centre for Emotional Health, Macquarie University; 2011.

This original manual, used as a control group manual, has been shown in a previous study to reduce symptoms of depression and anxiety in older adults:

Wuthrich VM, Rapee RM, Kangas M, Perini S. Randomized controlled trial of group cognitive behavioral therapy compared to a discussion group for co-morbid anxiety and depression in older adults. Psychol Med. 2016;46:785–95. https://doi.org/10.1017/S0033291715002251.

In the first half of each session, there is a weekly review in which participants freely exchange experiences, thoughts or feelings from the past week.

| **Session Nr.** | **Content of session** | **Discussion Topics** |
| --- | --- | --- |
| 1 | **Introduction, familiarisation** | Introduction to the programme's objectives and structure  familiarisation with each other, establishing group rules  perception exercises |
| 2 | **Depression symptoms** | E.g.: What symptoms of depression do you experience? In what situations do you notice them? |
| 3 | **Anxiety symptoms** | E.g.: What symptoms of anxiety do you experience? In what situations do you notice them? |
| 4 | **Retirement Part 1: Expectations, experiences & challenges** | E.g.: How long have you been retired? Is retirement what you imagined it would be? What has surprised you? |
| 5 | **Retirement Part 2: Values, work, responsibility in old age** | E.g.: Should people be allowed to continue working after retirement or should they even be expected to?  What does ‘work’ mean to you today, beyond gainful employment? |
| 6 | **Generational**  **Differences Part 1** | E.g.: What differences between younger and older people have you noticed (in life models, the school and education system, etc.)? |
| 7 | **Generational**  **Differences Part 2** | E.g.: What differences do you notice in the use of digital media, smartphones, the internet? What differences do you notice in communication and personal encounters? |
| 8 | **Animal Companionship** | E.g.: Is a pet an extended member of the family? What are the advantages and disadvantages of having a pet? |
| 9 | **Collections & Possessions Part 1** | E.g.: Do you have or have you ever had a collection? What did you collect or do you perhaps still collect today? |
| 10 | **Collections & Possessions Part 2** | E.g.: What problems do people have with downsizing or reducing their possessions? Why do you think people find it difficult to get rid of certain things? |
| 11 | **Holidays Part 1** | E.g.: What trips are you currently planning? |
| 12 | **Family** | E.g.: What does family mean to you today and who belongs to it? |
| 13 | **Social Changes Part 1** | E.g.: How do relationships change as we get older? For example, in a partnership through taking on caregiving responsibilities? In parent-child dynamics? |
| 14 | **Social Changes Part 2** | E.g.: How has technology changed forms of communication (telephone, mobile phone, social networks)? Does this promote or hinder social participation in old age? |
| 15 | **Hobbies Part 1** | E.g.: What exactly do we mean by a ‘hobby’? Why do people have hobbies? What role do hobbies play in everyday life? |
| 16 | **Hobbies Part 2** | E.g.: Are there any hobbies that you share with others? What does it take for a hobby to be truly enjoyable? |
| 17 | **Global Issues**  **Part 1** | E.g.: What does ‘globalization’ mean and where do we encounter it in everyday life? What advantages does global networking bring, and what challenges? |
| 18 | **Global Issues**  **Part 2** | E.g.: What comes to mind when you think of ‘everyday life in another country’? Have you ever lived anywhere else? |
| 19 | **Cooking & Cousin** | E.g.: What dishes do you like to eat/cook? What role does food play in your everyday life – is it more of a habit, a conscious decision or a moment of enjoyment? |
| 20 | **farewell** | closing party |
